# Autonomic Gating of Ripple Mode Selection at Sleep Onset

**DOI:** 10.1007/s12264-026-01635-0

**Published:** 2026-05-15

**Authors:** Mariana Toricelli, Sergio Tufik, Monica Levy Andersen

**Affiliations:** 1 Sleep Institute/Research Incentive Fund Association (AFIP), São Paulo, 04024-002 Brazil; 2 Department of Psychobiology, Federal University of São Paulo (UNIFESP), São Paulo, 04024-002 Brazil

Sleep-dependent memory consolidation relies on coordinated interactions between hippocampal replay, cortical oscillations, and brain state transitions that unfold across the sleep onset period and early non-rapid eye movement (NREM) sleep [1]. While substantial progress has been made in identifying the neuronal substrates of replay and its role in systems consolidation [2, 3], less attention has been paid to how global physiological states shape the conditions under which distinct replay patterns are expressed and effectively coupled to downstream consolidation processes. In particular, autonomic regulation during the wake-to-sleep transition has emerged as a key modulator of NREM sleep depth, oscillatory synchronization, and hippocampal cortical communication [4], yet its potential influence on the structure of hippocampal replay remains largely unexplored.

Castelli Manfredi and colleagues provide an elegant demonstration that hippocampal replay is not uniform, but instead emerges through 2 laminar-specific ripple modes, Radsink and LMsink, which differentially support the rapid consolidation of recent memories and the gradual integration of remote representations [5]. This distinction advances existing models in which the hippocampus balances plasticity and stability across offline brain states. We would like to suggest that the autonomic state at sleep onset may serve as an upstream regulator of the physiological context in which ripple modes are expressed and consolidated. A stronger version of this hypothesis is that autonomic state biases ripple mode expression itself. A more conservative version is that autonomic state chiefly biases the oscillatory and neuromodulatory conditions under which a given ripple mode can most effectively engage hippocampal cortical transfer. While the authors interpret ripple diversity mainly in terms of CA3 versus entorhinal drive, convergent evidence indicates that the transition into sleep is systematically shaped by sympathovagal balance, with consequences for NREM sleep depth and oscillatory coordination across hippocampo–thalamo–cortical circuits.

A well-established literature shows that heightened sympathetic arousal, as seen in stress, hypervigilance, anxiety, and insomnia, is associated with lighter NREM sleep, reduced slow-wave continuity, and altered cortical oscillatory entrainment [6]. In contrast, increased vagal tone promotes deeper NREM sleep, stronger slow oscillations, and enhanced spindle–slow oscillation coupling, a known facilitator of hippocampal–neocortical information transfer [7].

Recent mechanistic work strengthens this link between arousal-related autonomic tone and NREM sleep stability by identifying the locus coeruleus noradrenergic system as an organizer of infraslow NREM sleep substates [8]. Osorio Forero *et al*. showed in mice that infraslow, approximately 50-second fluctuations in locus coeruleus activity partition NREM sleep into alternating brain autonomic states: higher locus coeruleus activity induces a subcortical autonomic arousal state that facilitates cortical microarousals and fragments NREM sleep, whereas lower locus coeruleus activity creates permissive windows for NREM to REM sleep transitions [8]. Functionally, this alternation acts as a gatekeeper of the NREM/REM sleep cycle by creating discrete permissive troughs for REM sleep entry, and under REM sleep restriction, it limits REM sleep entries to at most one per approximately 50-second cycle [8]. A stimulus-enriched and stress-promoting wakefulness biased subsequent NREM sleep toward longer high activity epochs and shorter low activity epochs, resulting in more microarousal-driven fragmentation and delayed REM sleep onset [8]. Deng *et al*. further refine this framework by showing that consolidation-relevant hippocampal cortical communication during NREM sleep is temporally gated [9]. In mice, using a genetically encoded cAMP sensor with high temporal resolution, they identified infraslow cAMP oscillations that were synchronized across hippocampus and cortex and depended on norepinephrine β1 receptor signaling, with a narrow peak cAMP window during which hippocampal cortical interactions increased and hippocampal perturbation selectively impaired subsequent memory [9]. These findings offer a plausible molecular and temporal framework that complements the locus coeruleus state framework: autonomic and neuromodulatory settling during sleep onset may regulate the probability of entering consolidation permissive windows, thereby biasing when hippocampal replay is most effective at engaging cortical networks [9]. This temporal organization provides a plausible context for the more conservative component of our proposal, namely that distinct ripple modes may differ in their effectiveness across consolidation permissive windows. Whether the autonomic and neuromodulatory state also shifts the probability that specific ripple modes are generated remains an open question [9]. Together, these findings nominate a concrete neuromodulatory mechanism through which arousal-related neuromodulatory and autonomic dynamics at sleep onset can shape early NREM sleep stability and the oscillatory coordination that scaffolds hippocampal cortical communication. Across human studies, autonomic state is operationalized using ECG-derived measures (heart rate bursts and heart rate variability), time aligned to central NREM sleep events, enabling an event-level characterization of central autonomic coupling. Importantly, in humans, autonomic central coupling during NREM sleep has been linked to declarative memory consolidation: Naji *et al*. described autonomic central events in which transient heart rate bursts are preceded by increases in delta and sigma activity and slow oscillation density, followed by a surge in vagal activity, and the occurrence of these coordinated events predicts post-sleep improvement in declarative memory [10]. Consistent with this, the strength of slow oscillation spindle coupling during NREM sleep has been reported to scale with high-frequency heart rate variability, linking more precise NREM sleep coordination to higher parasympathetic tone [11]. Likewise, individualized temporal structure is a major determinant of when spindles occur across the night, highlighting that variability in spindle timing can be trait-like and not fully explained by conventional state descriptors such as sleep depth [12].

## This Leads to a Testable Hypothesis:

**Fig. 1:**
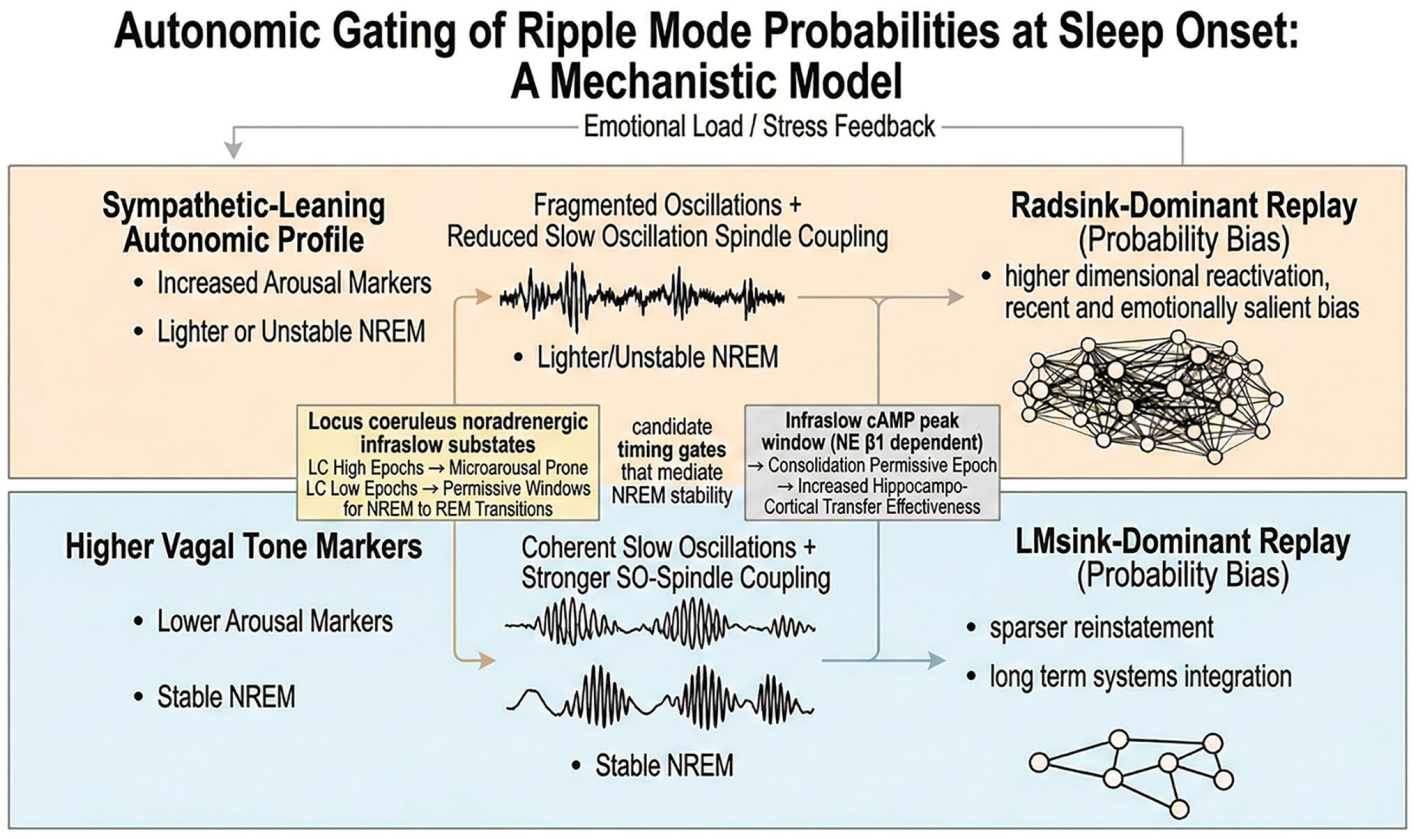
Autonomic shaping of ripple mode expression and consolidation effectiveness at sleep onset. Schematic illustrating a plausible, testable systems-level hypothesis in which arousal-related autonomic markers during the wake-to-sleep transition shape early NREM sleep stability and oscillatory coordination. The primary hypothesis is that these state variables define consolidation permissive versus non-permissive windows, thereby altering the effectiveness with which ripple-associated replay engages hippocampal cortical transfer. A secondary and more speculative hypothesis is that the same autonomic and oscillatory conditions may also bias the probability that distinct laminar-specific ripple modes are expressed. Sympathetic-leaning autonomic profiles with increased arousal markers are linked to lighter or unstable NREM sleep, fragmented oscillations, and reduced slow oscillation spindle coupling, conditions predicted to reduce integrative replay effectiveness and to favor stabilization of recent and emotionally salient information. Higher vagal tone markers with lower arousal markers are linked to stable NREM sleep, coherent slow oscillations, and stronger slow oscillation spindle coupling, conditions predicted to enhance integrative consolidation across distributed networks. Candidate timing mechanisms that may contribute to these state transitions include locus coeruleus noradrenergic infraslow substates, which regulate NREM sleep microarousability, and a norepinephrine β1-dependent infraslow cAMP peak window, which may define epochs of increased hippocampal cortical transfer effectiveness. The model can be tested by jointly quantifying sleep onset autonomic markers, microarousal density, slow oscillation spindle coupling strength, ripple mode classification by laminar current sink profiles, and memory outcomes

Mechanistically, we propose that sleep onset arousal may influence microarousability and thalamo cortical synchrony, thereby shaping the oscillatory context in which hippocampal replay unfolds. Our primary hypothesis is that arousal-related neuromodulatory and autonomic dynamics during the wake-to-sleep transition may influence early NREM sleep stability and oscillatory coordination, thereby altering the conditions under which ripple-associated replay can most effectively engage hippocampal cortical transfer and support consolidation. Specifically, sleep onset profiles characterized by higher sympathetic arousal markers and reduced vagal support are associated with more fragmented NREM sleep, greater microarousability, and weaker slow oscillation–spindle coordination, conditions predicted to favor Radsink, like higher dimensional reactivation that preferentially stabilizes recent and emotionally salient representations. In contrast, sleep onset profiles characterized by stronger vagal tone markers and lower arousal are linked to more stable NREM sleep, more coherent slow oscillations, and stronger network synchrony, conditions predicted to favor LMsink-like sparser reinstatement and integrative consolidation across distributed networks. A secondary and more speculative claim is that these same autonomic and oscillatory conditions may also shift the probability that distinct laminar ripple modes are expressed, with more arousal-prone epochs favoring Radsink-like dynamics and more stable epochs favoring LMsink-like dynamics. A key boundary condition is that ripple diversity in the original study was characterized in mice during stable NREM sleep, where autonomic state is comparatively constrained and does not systematically vary across sleep onset. In humans, by contrast, autonomic dynamics fluctuate substantially across the wake-to-sleep period, and this variability is accentuated in insomnia and affective disorders, which also show selective biases in sleep-dependent consolidation toward emotionally charged material and negative contextual associations [13]. These converging observations motivate the possibility that subtle shifts in early sleep microarchitecture and its coupled autonomic landscape may influence not only whether specific replay dynamics emerge, but also whether those dynamics occur within a consolidation-permissive context [14]. This framework makes concrete predictions. The model would be challenged if laminar ripple mode prevalence and reactivation dynamics remain invariant across large within-subject shifts in sleep onset autonomic markers and objective NREM sleep stability metrics, or if ripple modes vary without any corresponding change in oscillatory coordination. Conversely, simultaneous central and peripheral recordings across sleep onset, with ripple modes classified by laminar current sink profiles and NREM sleep stability quantified by microarousal density and slow oscillation spindle coupling, could reveal that periods of reduced microarousability and stronger oscillatory coordination coincide with windows in which LMsink like replay is preferentially expressed or preferentially coupled to hippocampal cortical transfer, whereas more arousal prone epochs bias replay toward Radsink like dynamics. We offer this perspective not to revise the authors’ central conclusions, but to propose that the autonomic landscape of early sleep may shape consolidation permissiveness at a systems level and may, under some conditions, also bias ripple mode expression, NREM sleep microarchitecture, and laminar ripple profiles during consolidation [7] (Fig. 1).
